# Supplementary material for: International testing and refinement of AI algorithms predicting acute leukemia subtypes from routine laboratory data
Source: Nat Commun. 2026 Mar 20;17:2649. doi: 10.1038/s41467-026-70584-z (PMC13004982; doi:10.1038/s41467-026-70584-z)
Supplement: Supplementary file 1 — Supplementary Information [file 41467_2026_70584_MOESM1_ESM.pdf]

Supplementary Information to

International testing and refinement of AI algorithms predicting acute leukemia subtypes from routine laboratory data

Amin T. Turki[1-3], Yi Fan[4], Alberto Hernández-Sánchez[5], Wellington Silva[6], Shaun Fleming[7], Koray Yalcin[8], Catharina H.M.J. Van Elssen[9], Yazan Madanat[10], Magdalena Karasek[11], Mahmoud Aljurf[12], Matteo G. Della Porta[13], Alexandra Martinez-Roca[14], Luca Guarnera[15], Katarina Steffen[16], Evangelia Antoniou[17], Maria M. Rivas[18], Deepak K Mishra[19], Ansgar T. Blum[2], Stephania Niry Manantsoa[20], Adeniyi Adiat[21], Amir Enshaei[22], Felicitas Thol[23], Maria Teresa Voso[15], Jia Chen[4], Tusneem Ahmed Elhassan[12], Anthony Moorman[22], María Belén Vidriales[5], Nina R. Neuendorff[2], Ahmet Koc[24], Pratyush Mishra[19], Dirk Strumberg[2], Roma S Fourmanov[9], Lukas Heine[3], Jens Kleesiek[3], Daniel Munárriz[14], Gianluca Asti[13], Mridula Mokoonlall[7], Marisa Kometas[10], Eduardo Rego[6], Rabea Mecklenbrauck[23], Marta Sobas[11], Depei Wu[4], Felix Nensa[3] and Merlin Engelke[3]

Author Affiliations:

1. Computational Hematology Lab, Institute for Artificial Intelligence in Medicine, University Hospital Essen, Essen, Germany
2. Department of Hematology and Oncology, University Hospital Marienhospital, Ruhr-University Bochum, Bochum, Germany
3. Institute for Artificial Intelligence in Medicine, University Hospital Essen, Essen, Germany
4. The First Affiliated Hospital of Soochow University, Suzhou, China
5. Department of Hematology, University Hospital of Salamanca, Spain
6. Hospital das Clinicas da Faculdade de Medicina da Universidade de Sao Paulo, Brazil
7. The Alfred Hospital, Melbourne, Australia
8. Bahcesehir University Medicalpark Goztepe Hospital, Department of Pediatric Hematology/Oncology, Istanbul, Turkey
9. Maastricht University Medical Center, Department of Internal Medicine, Division of Hematology & GROW research, Institute of Oncology and Reproduction, Maastricht, The Netherlands
10. University of Texas Southwestern, Dallas, USA
11. Department of Hematology, Cell Therapies and Internal Medicine, Wroclaw Medical University, Poland
12. King Faisal Specialist Hospital & Research Center, Riyadh, Saudi Arabia
13. IRCCS Humanitas Research Hospital & Humanitas University Milan, Milan, Italy
14. Department of Hematology, Hospital Clínic Barcelona, Barcelona, Spain
15. Tor Vergata University Roma, Rome, Italy
16. Central Laboratory Division, University Hospital Marienhospital, Ruhr-University Bochum, Bochum, Germany
17. Department of Pediatrics III, University Hospital Essen, Essen, Germany
18. Hospital Universitario Austral, Buenos-Aires, Argentina
19. Tata Medical Center, Kolkata, India
20. University Hospital of Antananarivo, Madagascar
21. Lagos University, Lagos, Nigeria
22. Leukaemia Research Cytogenetics Group, Translational and Clinical Research Institute, Newcastle University, Newcastle upon Tyne, United Kingdom
23. Department of Hematology, Hannover Medical School, Hannover, Germany
24. Marmara University Faculty of Medicine, Department of Pediatric Hematology and Oncology, Istanbul, Turkey

Supplementary Methods

Employed Python libraries

- FHIR-PYrate: Hosch R., Baldini G., Parmar V., et al. FHIR-PYrate: a data science friendly Python package to query FHIR servers. BMC Health Services Research. 2023;23:734. doi:10.1186/s12913-023-09498-1.

- NumPy: Harris CR., Millman KJ., van der Walt SJ., et al. Array programming with NumPy. Nature. 2020;585:357–362. doi:10.1038/s41586-020-2649-2.

- SciPy: Virtanen P., Gommers R., Oliphant TE., et al. SciPy 1.0: Fundamental Algorithms for Scientific Computing in Python. Nature Methods. 2020;17(3):261–272. doi:10.1038/s41592-019-0686-2.

- Pandas: McKinney W. Data structures for statistical computing in Python. In: Proceedings of the 9th Python in Science Conference. 2010;51–56. doi:10.25080/Majora-92bf1922-00a.

- Matplotlib: Hunter JD. Matplotlib: A 2D Graphics Environment. Computing in Science & Engineering. 2007;9(3):90–95. doi:10.1109/MCSE.2007.55.

- Seaborn: Waskom ML. seaborn: statistical data visualization. Journal of Open Source Software. 2021;6(60):3021. doi:10.21105/joss.03021.

- PyYAML: PyYAML Documentation. Available at: https://pyyaml.org/. Accessed April 2, 2025.

- Scikit-learn: Pedregosa F., Varoquaux G., Gramfort A., et al. Scikit-learn: Machine Learning in Python. Journal of Machine Learning Research. 2011;12:2825–2830.

- SHAP: Lundberg SM., Erion G., Chen H., et al. From local explanations to global understanding with explainable AI for trees. Nature Machine Intelligence. 2020;2:56–67. doi:10.1038/s42256-019-0138-9.

- Weights & Biases (wandb): Weights & Biases Documentation. Available at: https://docs.wandb.ai/. Accessed April 2, 2025.

- python-dotenv: python-dotenv - Read key-value pairs from a .env file and set them as environment variables. Available at: https://github.com/theskumar/python-dotenv. Accessed April 2, 2025.

- XGBoost: Chen T., Guestrin C. XGBoost: A Scalable Tree Boosting System. In: Proceedings of the 22nd ACM SIGKDD International Conference on Knowledge Discovery and Data Mining. 2016;785–794. doi:10.1145/2939672.2939785.

Data requirements for implementing the preprocessing pipeline

The preprocessing pipeline may be implemented at different levels. The basic preprocessing pipeline requires the same minimal data required for running the algorithm as detailed in the main methods (i.e. total White Blood Cell Count (WBCWBC; 10^9 per L), monocyte and lymphocyte counts (10^9 per L), platelets (10^9 per L), Mean Corpuscular Volume (MCV; fL), Mean Corpuscular Hemoglobin Concentration (MCHC; g/L),), monocyte and lymphocyte counts, platelets, Mean Corpuscular Volume (MCV), Mean Corpuscular Hemoglobin Concentration (MCHC) as well as Lactate Dehydrogenase (LDH, IU/L), fibrinogen (g/L,) and prothrombin time (in %)). For preparing an extended preprocessing pipeline, further laboratory features were added, in particular neutrophil granulocytes in absolute counts and in %, lymphocytes in % that support the exclusion of differential diagnosis before starting the acute leukemia algorithm (Supplementary Table 1).

Supplementary Figure index

Supplementary Figure 1. AI methodology overview. Visualization of the AI pipeline and methods. The steps in data preparation, preprocessing, model evaluation, analysis and refinement are summarized in a stepwise approach. The figure was created with Biorender.

Supplementary Figure 2. STROBE diagram of cohort selection. The figure was created with Biorender.

Supplementary Figure 3. Adult cohorts' metrics. **a** Comparison of algorithm performance using the F1 score per cutoff and diagnosis. Centers are ranked according to the F1 score for AML from the highest value to the lowest. Left: No cutoff. Middle: Overall cutoff. Right: Confident cutoff **b** Adult patients' XGBoost false negative predictions ranked by center. Bars are given as median ± interquartile range. Whiskers correspond to 1.5x interquartile range from box bounds

Supplementary Figure 4. Boxplot comparison of feature distributions between patients with true (positive and negative combined) and false (negative and positive combined) predictions. Results are aggregated for each class, AML, ALL, APL. Left row: all patients. Middle row: patients with true (positive and negative combined) predictions. Right row: Patients with false (positive and negative combined) predictions. Bars are given as median ± interquartile range. Middle line shows median, upper box margin 75%, lower box margin 25%. Whiskers correspond to 1.5x interquartile range from box bounds.

Supplementary Figure 5. Scatterplot of feature distributions sorted by feature, no filtering. Individual patients are colored according to their true class. AML, red; APL, green; ALL, blue

Supplementary Figure 6 Comparison of the algorithm performance (AUROC) per centrally tested center before and after out of distribution detector.

Supplementary Figure 7. Results on the pediatric cohort predictions **a** Pretrained model explainability by SHAPley values in the pediatric cohorts for AML, APL, and ALL supports the important of distinct features in each prediction setting. Features are ranked from top to bottom according to their importance (right side favors the subtype prediction). Each laboratory parameter is colored according to its value with higher values shown in red. **b** True positive predictions for the pretrained model tested on pediatric patients per center. AML (red), APL (green) and ALL (blue). Boxplots are only shown for classes reaching minimum numbers per center. Bars are given as median ± interquartile range. **c** False negative predictions for AML, APL and ALL. **d** Pediatric patients' pretrained model AUROC metrics shown for each of the tested sites for AML (red), APL (green line) and ALL (blue). Patients from Riyadh underwent local testing.

Supplementary Figure 8. Per decade comparison of accuracy metrics (AUROC) in adult and pediatric patients per Leukemia subtype. Each stratum refers to one age subset. Left: AML, age per decades 0-9, 10-19 etc. Middle: APL. Right: ALL.

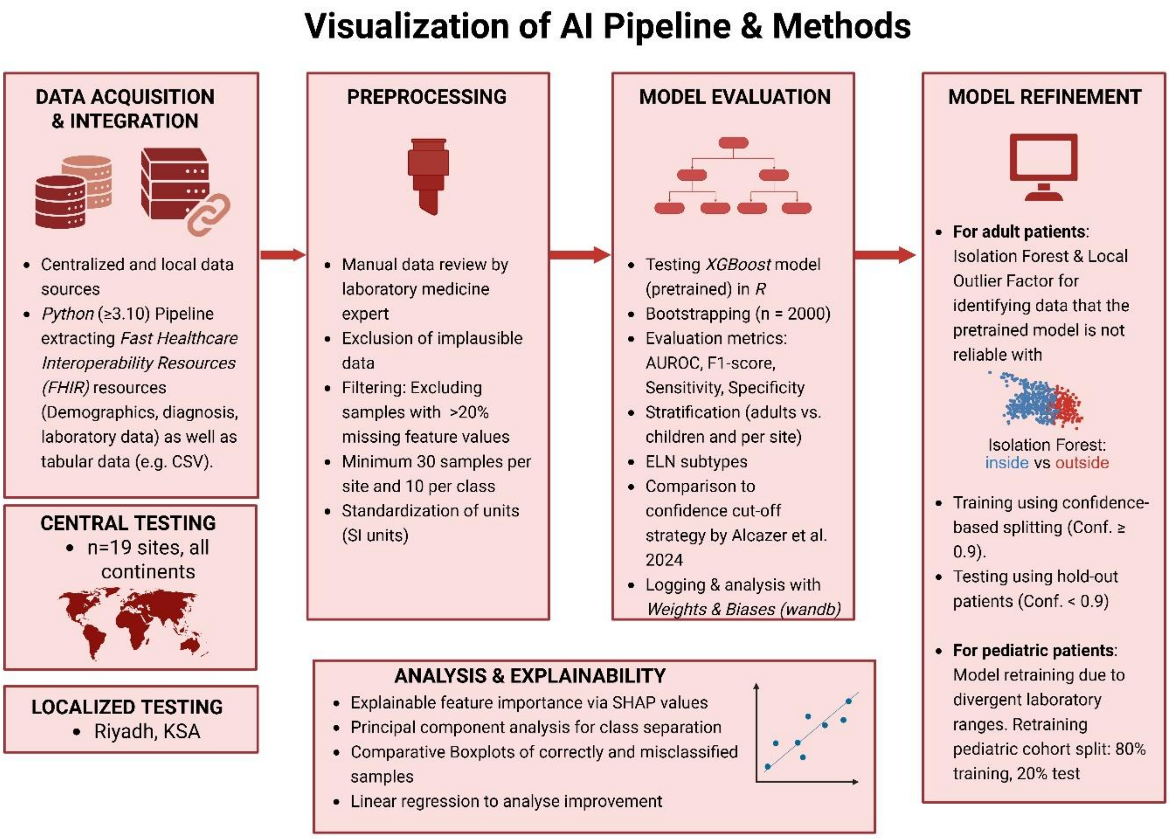

Supplementary Figure 1. AI methodology overview. Visualization of the AI pipeline and methods. The steps in data preparation, preprocessing, model evaluation, analysis and refinement are summarized in a stepwise approach. The figure was created with Biorender.

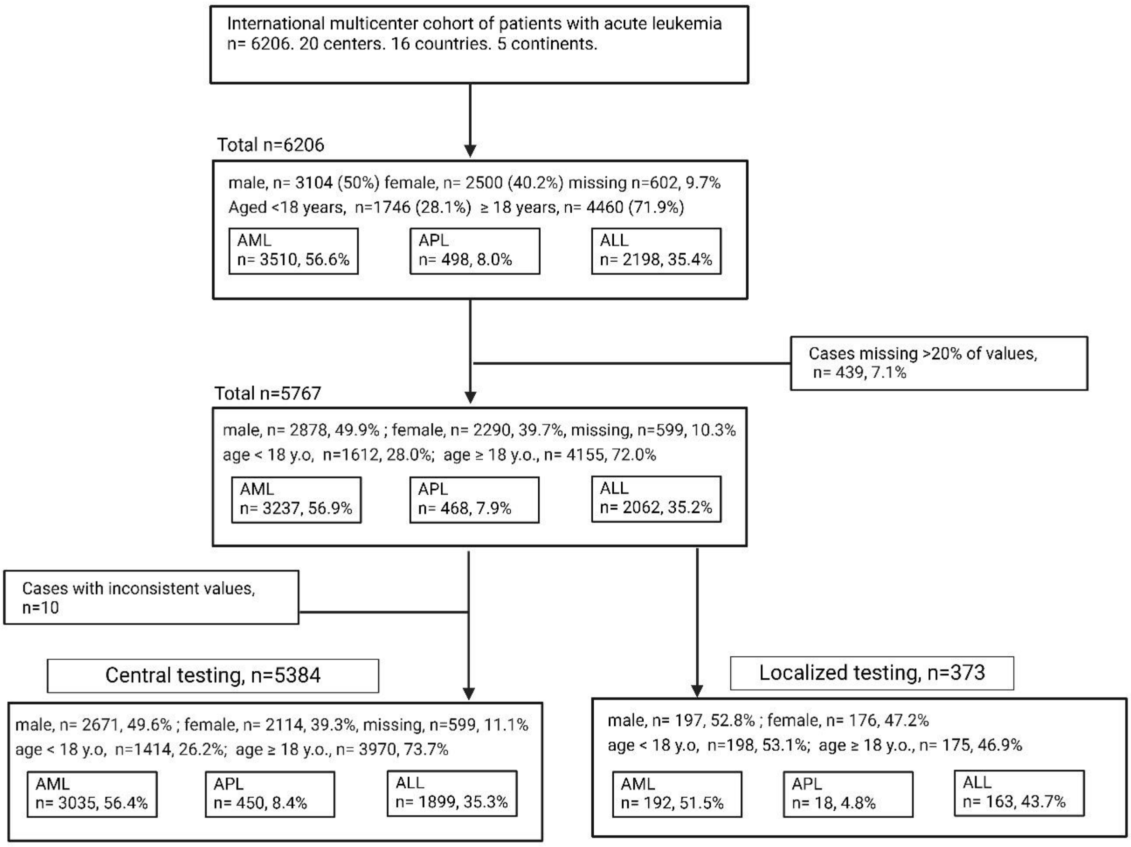

Supplementary Figure 2. STROBE diagram of cohort selection. The figure was created with Biorender.

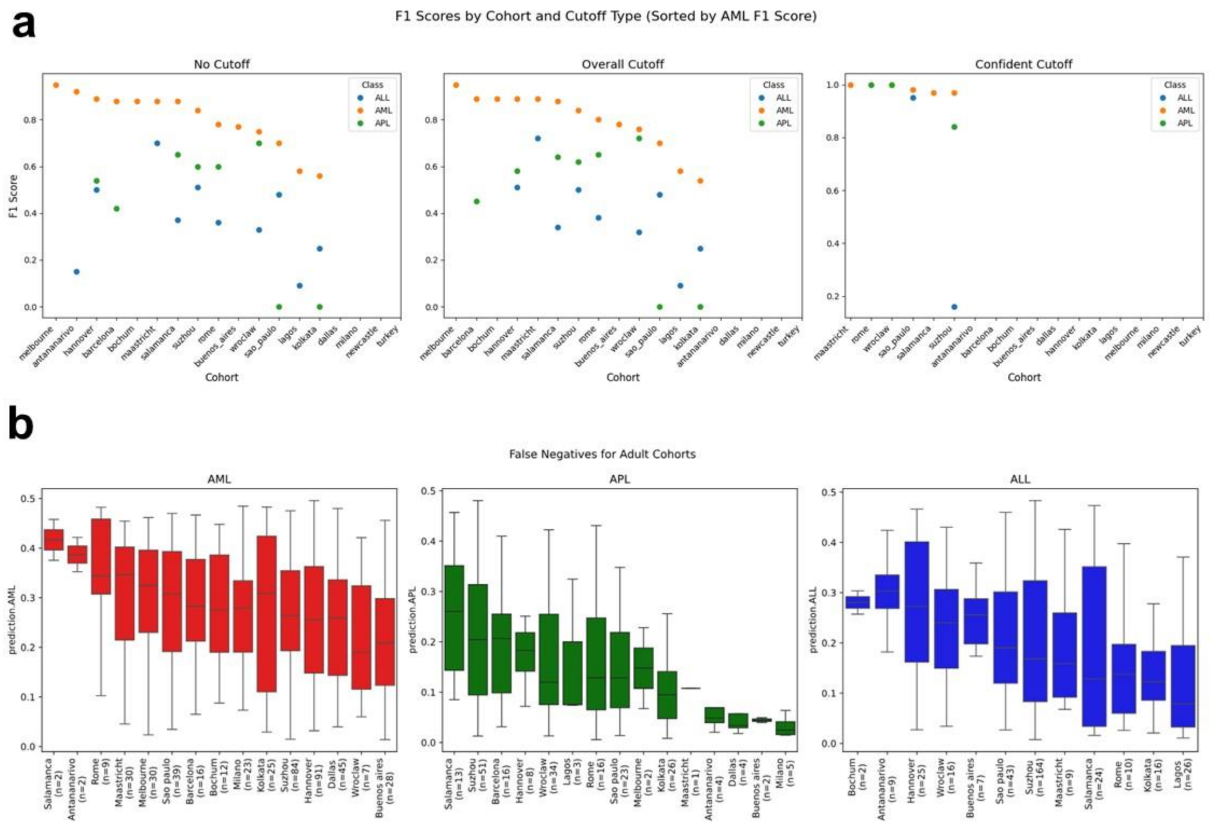

Supplementary Figure 3. Adult cohorts' metrics. **a** Comparison of algorithm performance using the F1 score per cutoff and diagnosis. Centers are ranked according to the F1 score for AML from the highest value to the lowest. Left: No cutoff. Middle: Overall cutoff. Right: Confident cutoff **b** Adult patients' XGBoost false negative predictions ranked by center. Bars are given as median ± interquartile range. Whiskers correspond to 1.5x interquartile range from box bounds

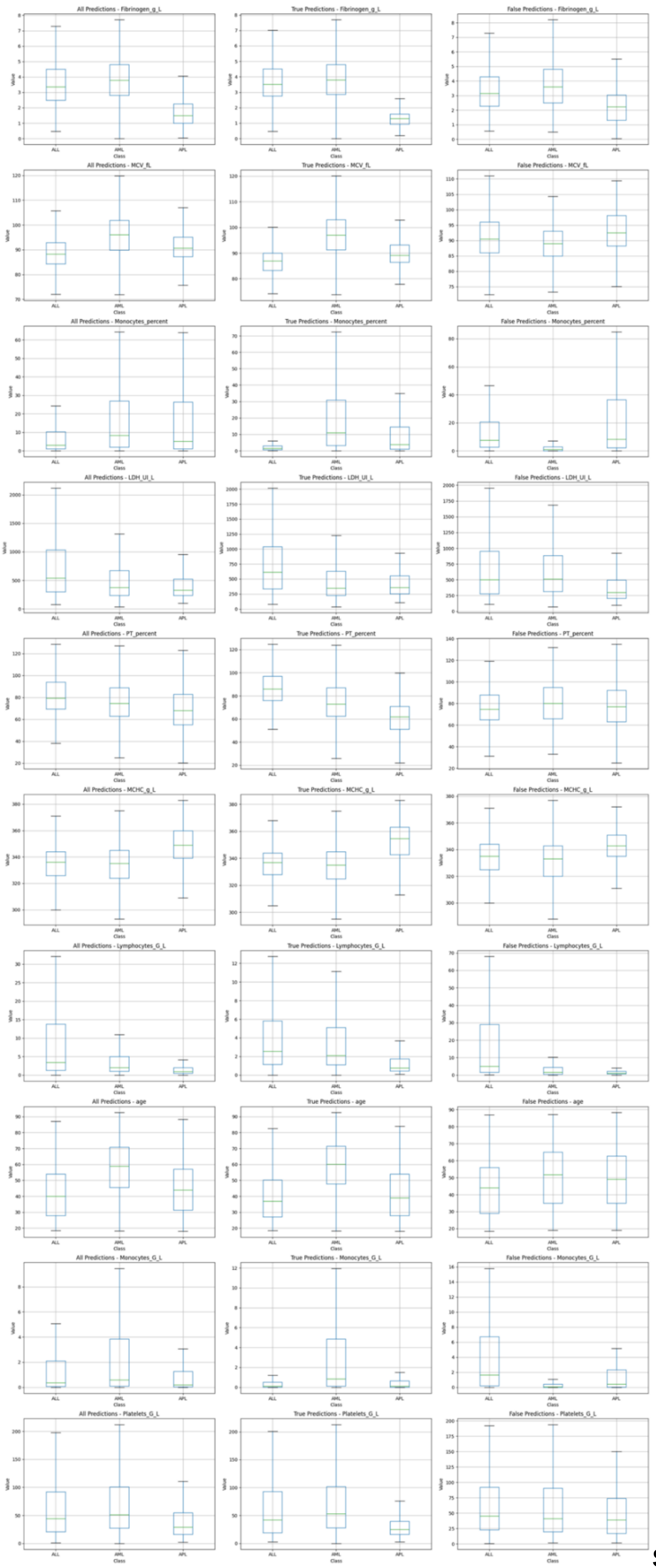

Supplementary Figure 4. Boxplot comparison of feature distributions between patients with true (positive and negative combined) and false (negative and positive combined) predictions. Results are aggregated

for each class, AML, ALL, APL. Left row: all patients. Middle row: patients with true (positive and negative combined) predictions. Right row: Patients with false (positive and negative combined) predictions. Bars are given as median ± interquartile range. Middle line shows median, upper box margin 75%, lower box margin 25%. Whiskers correspond to 1.5x interquartile range from box bounds.

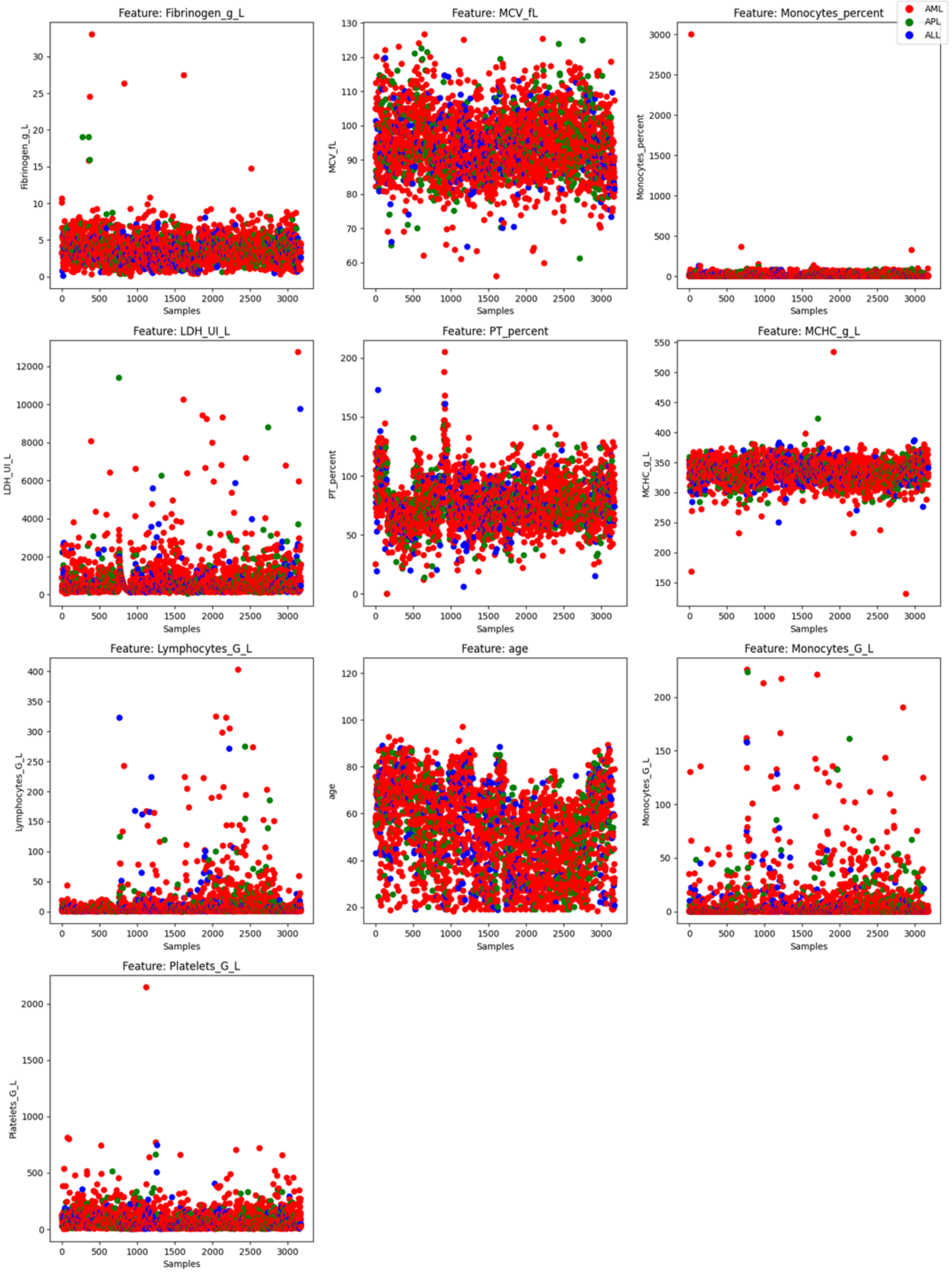

Supplementary Figure 5. Scatterplot of feature distributions sorted by feature, no filtering. Individual patients are colored according to their true class. AML, red; APL, green; ALL, blue

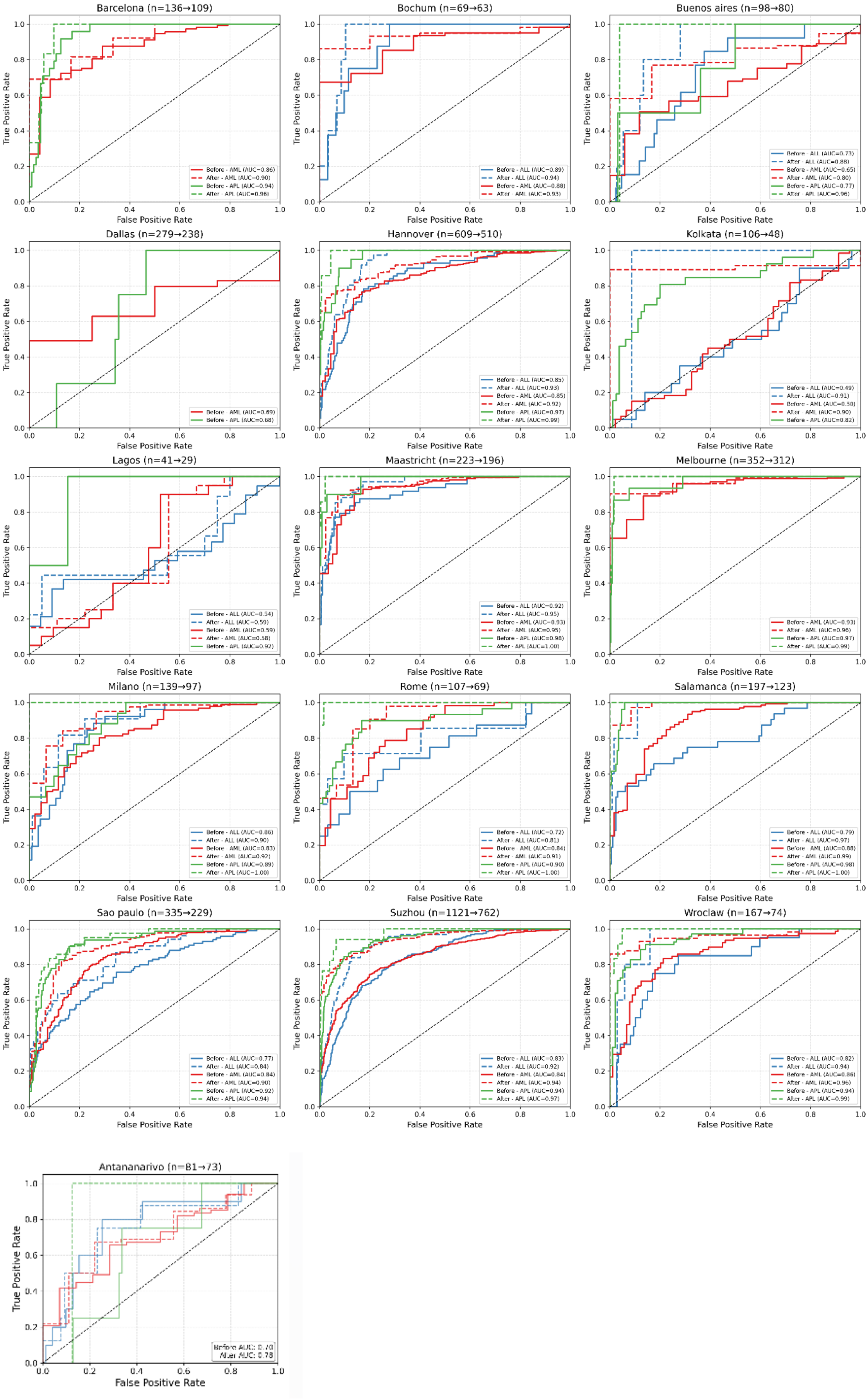

Supplementary Figure 6 Comparison of the algorithm performance (AUROC) per centrally tested center before and after out of distribution detector.

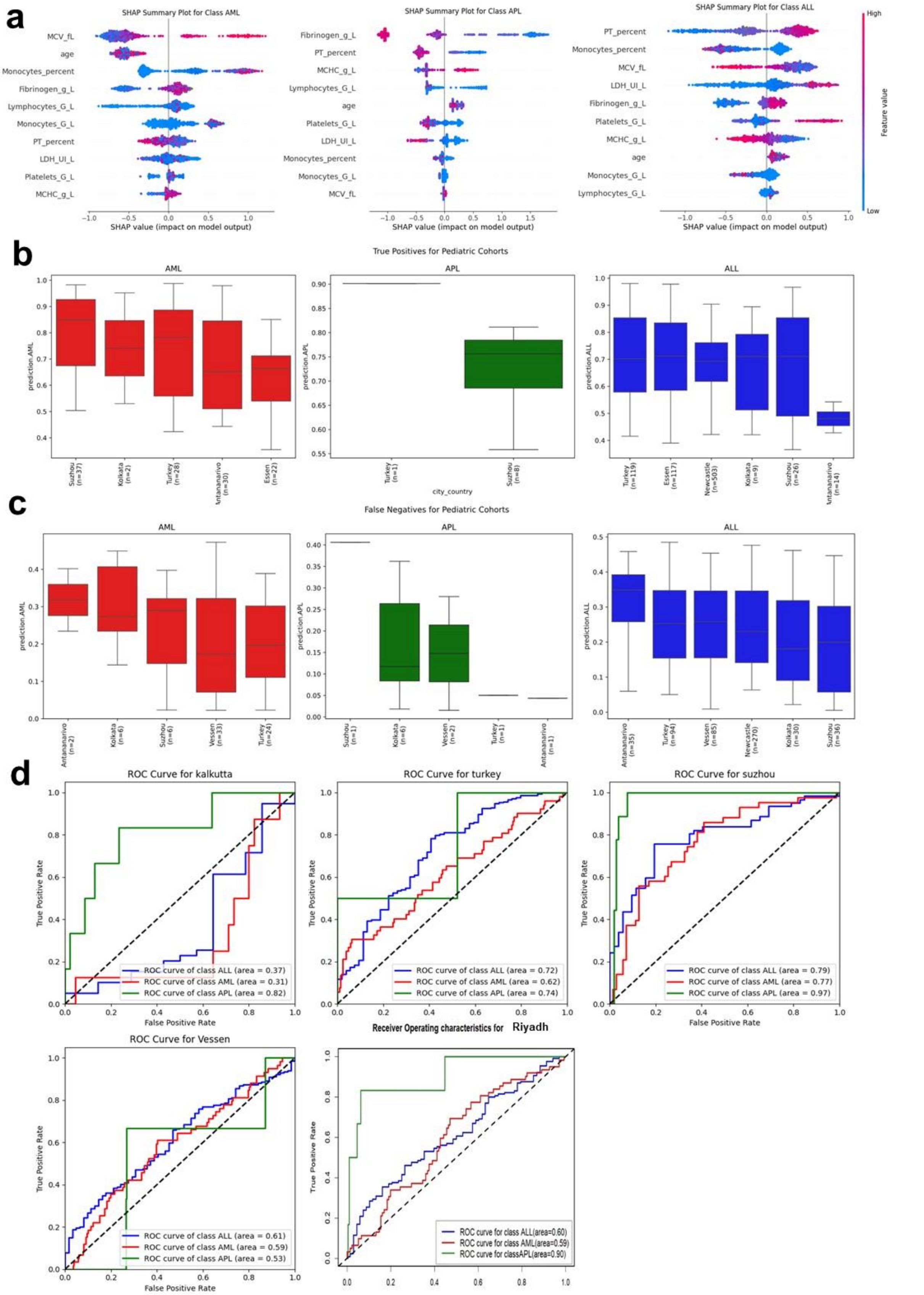

Supplementary Figure 7. Results on the pediatric cohort predictions **a** Pretrained model explainability by SHAPley values in the pediatric cohorts for AML, APL, and ALL supports the important of distinct features in each prediction setting. Features are ranked from top to bottom according to their importance (right side favors the subtype prediction). Each laboratory

parameter is colored according to its value with higher values shown in red. **b** True positive predictions for the pretrained model tested on pediatric patients per center. AML (red), APL (green) and ALL (blue). Boxplots are only shown for classes reaching minimum numbers per center. Bars are given as median ± interquartile range. **c** False negative predictions for AML, APL and ALL. **d** Pediatric patients' pretrained model AUROC metrics shown for each of the tested sites for AML (red), APL (green line) and ALL (blue). Patients from Riyadh underwent local testing.

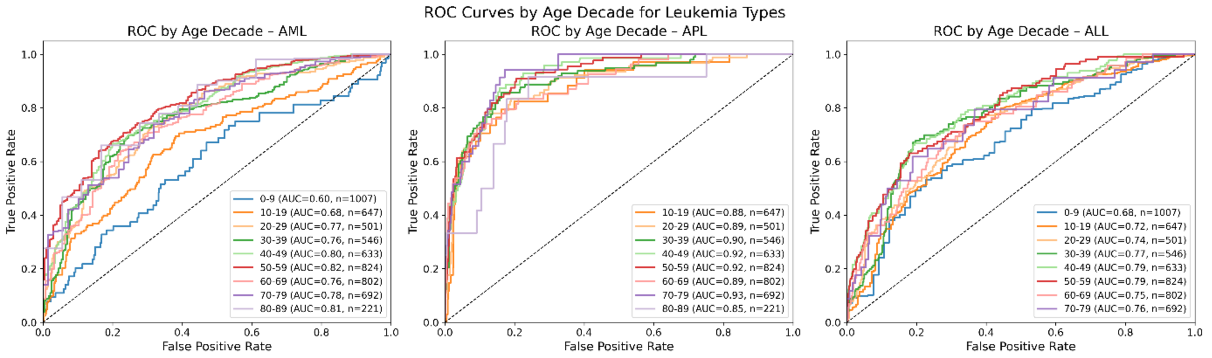

Supplementary Figure 8. Per decade comparison of accuracy metrics (AUROC) in adult and pediatric patients per Leukemia subtype. Each stratum refers to one age subset. Left: AML, age per decades 0-9, 10-19 etc. Middle: APL. Right: ALL.

Supplementary Tables index

Supplementary Table 1. Pipeline feature list and units.

Supplementary Table 2. Participating countries ranked according to United Nations Human Development Index (HDI)

Supplementary Table 3. Adult cohort baseline characteristics. The global cohort is described for baseline characteristics, including laboratory features. a-c) Centrally tested cohorts (n=19) sorted by AML, APL and ALL. d) Locally tested adult cohort from Riyadh.

Supplementary Table 4. Results of comparative experiments challenging the pipeline with differential diagnoses of acute leukemia. The experiments included patients with malignant conditions such as myelodysplasic syndromes, chronic lymphocytic leukemia, chronic myeloid leukemia, myeloproliferative neoplasms, mantle cell lymphoma, chronic myelomonocytic leukemia, juvenile myelomonocytic leukemia, but also those with reactive conditions such as vitamin B12 deficiency, folate deficiency, TTP, sepsis, parvovirus B19 infection, EBV infection, SARS-CoV-2.

Supplementary Table 5. Per decade comparison of accuracy metrics in adult and pediatric patients per leukemia subtype.

Supplementary Table 1. Feature list

a: Essential features for XGB model testing

complete blood cell (CBC) count:	
White blood cell count (total)	g/L
Monocyte counts	g/L
Lymphocyte counts	g/L
Platelet count	g/L
Mean Corpuscular Volume	fL
Mean Corpuscular Hemoglobin Concentration (MCHC; g/L)	g/L
Blood chemistry*:*	
Lactate Dehydrogenase	IU/L
Coagulation	
Fibrinogen	g/L
Prothrombin time	Percent
Age	years

b: Additional features for model pipeline preprocessing

Neutrophil granulocytes absolute counts (Required for pipeline)	g/L
Neutrophil granulocytes % (Required for preprocessing pipeline)	Percentage of WBC
Lymphocytes in % (Required for preprocessing pipeline)	Percentage of WBC

Supplementary Table 2. Participating countries ranked according to United Nations Human Development Index (HDI)

HDI rank	Country	United Nations Human Development Index (HDI)	UN/World Bank income category
1	Highest HDI	0.967	High income
7	Germany	0.950	High income
10	Australia	0.946	High income
10	Netherlands	0.946	High income
15	United Kingdom	0.940	High income
20	United States	0.927	High income
27	Spain	0.911	High income
30	Italy	0.906	High income
36	Poland	0.881	High income
40	Saudi Arabia	0.875	High income
45	Türkiye	0.855	Upper middle income
48	Argentina	0.849	Upper middle income
75	China	0.788	Upper middle income
89	Brazil	0.760	Upper middle income
134	India	0.644	Lower middle income
161	Nigeria	0.548	Lower middle income
177	Madagascar	0.487	Low income
193	Lowest HDI	0.380	Low income

Human Development Index (HDI): A composite index measuring average achievement in three basic dimensions of human development—a long and healthy life, knowledge and a decent standard of living. See Technical note 1 at http://hdr.undp.org/sites/default/files/hdr2023_technical_notes.pdf for details on how the HDI is calculated.

**UN HDI statistical References**	
World average HDI	0.739
Very high human development	0.902
High human development	0.764
Medium human development	0.640
Low human development	0.517

Supplementary Table 3. Adult cohort characteristics as feature distributions. The global cohort is described for baseline characteristics and laboratory feature distributions.

a) AML patients (central testing)

	Age	MCV (fl)	PT %	LDH (IU/l)	MCHC (g/l)	WBC (g/l)	Fibrinogen (g/l)	Monocytes (g/l)	Platelets (g/l)	Lymphocytes (g/l)
N	3121	3106	2995.00	2971	3052.00	3119.00	2474.00	2961.00	3118.00	2998.00
**Mean**	57.18	96.32	75.09	585.44	334.10	32.93	3.94	6.48	78.95	6.26
**Std**	17.01	20.76	23.20	687.71	21.73	56.18	1.76	18.94	94.55	16.84
**Min**	18.24	56.00	0.00	36.00	131.00	0.02	0.00	0.00	0.07	0.00
**25%**	45.00	89.80	63.00	236.80	324.00	2.70	2.81	0.10	27.00	1.02
**50%**	59.00	95.85	74.30	373.00	335.00	9.40	3.79	0.57	50.00	2.05
**75%**	70.67	101.80	88.65	670.00	345.00	38.39	4.80	3.77	100.00	5.01
**Max**	121.44	1114.40	172.79	11396	1000.00	556.44	33.00	225.58	2146.00	275.00

b) APL Patients (central testing)

	Age	MCV (fl)	PT %	LDH (IU/l)	MCHC (g/l)	WBC (g/l)	Fibrinogen (g/l)	Monocytes (g/l)	Platelets (g/l)	Lymphocytes (g/l)
**n**	448.00	448.00	444.00	428.00	442.00	448.00	439.00	416.00	447.00	423.00
**mean**	45.52	91.18	71.24	471.65	347.71	16.10	1.83	2.96	43.36	2.40
**std**	17.09	6.75	25.18	475.48	16.56	27.65	1.64	10.46	43.30	5.88
**min**	18.11	63.70	0.00	96.10	282.00	0.20	0.05	0.00	2.00	0.00
**25%**	31.00	87.20	55.14	240.25	339.00	1.20	1.01	0.04	16.50	0.55
**50%**	44.00	90.60	68.00	327.00	349.00	3.46	1.50	0.20	29.00	0.94
**75%**	57.13	95.10	82.78	527.00	360.00	18.38	2.26	1.30	52.50	2.04
**max**	97.00	114.60	205.00	6249.00	423.00	205.00	26.30	129.20	275.00	83.77

c) ALL Patients (central testing)

	Age	MCV (fl)	PT %	LDH (IU/l)	MCHC (g/l)	WBC (g/l)	Fibrinogen (g/l)	Monocytes (g/l)	Platelets (g/l)	Lymphocytes (g/l)
n	661.00	656.00	634.00	623.00	631.00	661.00	581.00	633.00	661.0	640.00
**mean**	41.71	88.99	81.52	986.54	335.36	49.46	3.61	4.82	72.9	21.77
**std**	16.18	7.39	17.93	1399.00	19.11	86.92	1.88	15.48	84.7	52.31
**min**	18.36	59.80	21.97	78.00	232.00	0.50	0.46	0.00	1.0	0.00
**25%**	28.00	84.10	69.50	299.50	326.00	4.30	2.48	0.06	21.0	1.34
**50%**	40.00	88.30	79.00	542.00	336.00	14.30	3.35	0.32	43.0	3.41
**75%**	54.00	92.93	93.83	1032.30	344.00	51.94	4.50	2.09	92.0	13.57
**max**	87.00	125.30	143.00	12753.00	412.00	704.81	27.43	217.05	771.0	402.80

d) Localized testing cohort characteristics (Riyadh adults)

		Age	MCV (fl)	PT %	LDH (IU/l)	MCHC (g/l)	WBC (g/l)	Fibrinogen (g/l)	Monocytes (g/l)	Platelets (g/l)	Lymphocytes (g/l)
N	Valid	175	175	172	175	160	175	147	38	175	153
	Missing	0		3	0	15	0	28	137	0	22
Mean		42.434	90.516	86.430	546.068	330.53	28.644	4.1434	4.1434	81.7371	3.6555
Median		40.000	89.600	87.000	404.000	331.00	7.8300	4.1300	4.1300	50.0000	1.5700
Std. Deviation		16.289	7.7349	11.915	448.383	15.207	48.631	1.64897	1.64897	108.432	6.0370

Supplementary Table 4. Results of comparative experiments challenging the pipeline with differential diagnoses of acute leukemia (n=341). Patient data was obtained from the participating centers and from public data (sources at bottom). The experiments included patients with malignant conditions such as myelodysplasic syndromes, chronic lymphocytic leukemia, chronic myeloid leukemia, myeloproliferative neoplasms, mantle cell lymphoma, chronic myelomonocytic leukemia, juvenile myelomonocytic leukemia, but also those with reactive conditions such as vitamin B12 deficiency, folate deficiency, TTP, sepsis, parvovirus B19 infection, EBV infection, SARS-CoV2.

Category	Percentage	Out of distribution, *n*	Total, *n*
Leukocytes below normal	78.4%	40	51
Leukocytes above normal	87.9%	109	124
Leukocytes within normal	90.4%	150	166
Diagnosis subsets			
Malignancies			
Chronic lymphocytic leukemia	81.3%	26	32
Chronic myeloid leukemia	94.7%	18	19
Juvenile myelomonocytic leukemia	100%	2	2
Lymphoma	62.5%	5	8
Myelodysplastic syndromes	60.0%	6	10
Myeloproliferative neoplasms	87.5%	7	8
Non-malignant disorders			
Vitamin B12 deficiency	71.4%	10	14
SARS-CoV2	93.7%	118	126
Sepsis	90.4%	103	114

Others: Individual cases (each n= 1) with folate deficiency, PNH, TTP, parvovirus B19 infection and EBV infection were not reported as separate entities but are included in the overall statistics.

Sources of public sample data in this table:

Ordoñez-Avila R, Parraga-Alava J, Hormaza JM, Vaca-Cárdenas L, Portmann E, Terán L, Dorn M. CBCovid19EC: A dataset complete blood count and PCR test for COVID-19 detection in Ecuadorian population. *Data Brief*. 2023 Mar 1;47:109016. doi: 10.1016/j.dib.2023.109016.

Johnson, A., Pollard, T., Shen, L. et al. MIMIC-III, a freely accessible critical care database. *Sci Data* 3, 160035 (2016). https://doi.org/10.1038/sdata.2016.35

Supplementary Table 5. Per decade comparison of accuracy metrics in adult and pediatric patients per Leukemia subtype

Age (years)	ALL	AML	APL
0–9	0.65	0.56	n/a
10–19	0.74	0.69	0.87
20–29	0.74	0.78	0.89
30–39	0.78	0.77	0.91
40–49	0.80	0.81	0.93
50–59	0.80	0.83	0.93
60–69	0.75	0.76	0.89
70–79	0.75	0.79	0.94
80–89	n/a	0.78	0.85

Abbreviations: 'n/a', no available AUC for that age/disease subgroup combination.
