# Supplementary material for: International testing and refinement of AI algorithms predicting acute leukemia subtypes from routine laboratory data
Source: Nat Commun. 2026 Mar 20;17:2649. doi: 10.1038/s41467-026-70584-z (PMC13004982; doi:10.1038/s41467-026-70584-z)
Supplement: Supplementary file 3 — Description of Additional Supplementary Files [file 41467_2026_70584_MOESM3_ESM.pdf]

Description of Additional Supplementary Files

Supplementary Data 1. Bootstrapped algorithm performance metrics in adult leukemia patients using the pretrained model. Performance metrics (AUC, accuracy, precision, recall, F1 score) are detailed by disease and by center and divided into "no cutoff" and "confident cutoff" predictions.

Supplementary Data 2. Comparing the feature value distribution of the centrally tested adult cohort to the feature distribution of the French adult cohort the algorithm was developed on.

Supplementary Data 3. Comparing the feature value distribution of the central pediatric cohort to the feature distribution of the French adult cohort, which the pretrained algorithm was developed on.

Supplementary Data 4. Bootstrapped algorithm performance metrics in pediatric leukemia patients using the pretrained model. Performance metrics (AUC, accuracy, precision, recall, F1 score) are detailed by disease and by center and divided into "no cutoff" and "confident cutoff" predictions.
